# Circulating Interleukin-22 in Patients with Acute Myocardial Infarction Undergoing Primary Percutaneous Coronary Intervention

**DOI:** 10.3390/jcm13174971

**Published:** 2024-08-23

**Authors:** Augusto Ferreira Correia, Carolina Gomes Cavalcanti de Oliveira, Dinaldo Cavalcanti de Oliveira, Michelly Cristina Pereira, Flavio Alisson Carvalho, Estevão Campos Carvalho Martins, Dinaldo Cavalcanti de Oliveira

**Affiliations:** 1 Internal Medicine Department, Cardiology Division, Federal University of Pernambuco, Recife 50670-901, Brazil; augusto.fcorreia@gmail.com (A.F.C.); michelly.pereira@ufpe.br (M.C.P.); 2 Cardiology Emergency Room of Pernambuco, University of Pernambuco, Recife 52010-010, Brazil; 3 Internal Medicine Department, Maurício de Nassau University, Recife 51021-140, Brazil; carolgoliveira00@gmail.com; 4 Internal Medicine Department, Faculty of Medicine of Sertão, Arcoverde 56512-670, Brazil; dinaldo1107@hotmail.com; 5 Mestre Vitalino Hospital, Caruaru 55015-901, Brazil; flavioalison@cardiol.br; 6 Galeão Air Force Hospital, Rio de Janeiro 21941-353, Brazil; decamposmartins.estevao@gmail.com

**Keywords:** IL-22, STEMI, ACS, atherosclerosis

## Abstract

Acute coronary syndrome (ACS) represents an important clinical manifestation of coronary artery disease (CAD) and is characterized by a particularly poor prognosis. Myocardial reperfusion through primary percutaneous coronary intervention (PPCI) is imperative in the event of acute ST elevation myocardial infarction (STEMI). Interleukin-22 (IL-22) regulates immune and inflammatory responses. This interleukin has been described in the scenario of the CAD, but there are no data in patients with STEMI undergoing PPCI. **Objectives**: The goals of this study were to investigate the differences in circulating IL-22 levels between patients with STEMI undergoing PPCI and healthy controls and to determine whether these differences were associated with the culprit coronary artery, door-to-balloon time (DBT), final angiographic result, CAD classification, and presence of diabetes mellitus (DM). **Methods**: A total of 280 participants were recruited, comprising 210 STEMI cases and 70 healthy controls. Participants underwent clinical and angiographic evaluations, and serum IL-22 levels were measured using an enzyme-linked immunosorbent assay (ELISA). Data analysis was performed using the Mann–Whitney and Fisher tests, with *p* < 0.05 indicating significance. **Results**: Serum IL-22 levels were lower in cases (149.63, 84.99–294.56) than in the controls (482.67, 344.33–641.00); *p* < 0.001. Lower IL-22 levels were associated with the right coronary artery (RCA) (144.57, 70.84–242.43; 146.00, 63.60–279.67; 191.71, 121.80–388.97); *p* = 0.033. IL-22 was lower with shorter DBT (≤60 min, 106.00, 49.60–171.71; >60 min, 153.00, 88.86–313.60); *p* = 0.043. **Conclusions**: IL-22 levels were significantly lower in patients with STEMI than in healthy controls.

## 1. Introduction

Cardiovascular disease (CVD) is the leading cause of death worldwide, and it has a significant impact on productivity and quality of life [1]. Atherosclerosis, a key contributor to CVD, is a chronic inflammatory disease characterized by endothelial dysfunction. In conjunction with other pathological events, this dysfunction leads to the formation of atheromas [2]. This is the pathophysiological cause of coronary artery disease (CAD), which can present as chronic coronary syndrome (CCS) or acute coronary syndrome (ACS) depending on the morphology and inflammatory status of the atheromas [3]. Acute ST elevation myocardial infarction (STEMI) accounts for 38% of ACS cases and is associated with the highest mortality rates [4]. STEMI often exhibits a completely occluded coronary artery, and primary percutaneous coronary intervention (PPCI) is the gold standard for reopening the vessel. Evidence has demonstrated that this approach reduces mortality and enhances patient prognosis [5,6].

IL-22 belongs to the IL-10 family and plays diverse roles in regulating cell growth and differentiation and in modulating immunological and inflammatory responses [7,8]. T helper cells type 22 (Th22) are the primary IL-22 producers. IL-22 exerts its effects by binding to a heterodimeric receptor complex composed of the primary IL-22R1 receptor and the IL-10R2 coreceptor. This binding induces intracellular signaling through the Janus kinase/signal transducer and activator of transcription (JAK/STAT) pathway [7,8,9]. In addition to the cell surface IL-22 receptor complex, there is a soluble IL-22-binding protein (IL-22BP) that demonstrates higher affinity and specificity for IL-22, making it an effective competitive inhibitor of IL-22 in vitro [10,11].

Interactions between IL-22 and its receptors trigger the production of adhesion molecules by endothelial cells, including intercellular adhesion molecule 1 (ICAM-1) and vascular cell adhesion molecule 1 (VCAM-1), which recruit monocytes [7,8]. IL-22 also acts in the regulation and proliferation of vascular smooth muscle cells, increasing atheroma growth [12]. These data show that IL-22 may affect atheroma development and instability because the activation of the signal transducer and activator of transcription 3 (STAT3) protein is directly related to leukocyte activation, the progression of endothelial dysfunction, and an intensified inflammatory cascade [13]. Nonetheless, the role of IL-22 in STEMI is still unclear due to the scarce literature on this topic.

Thus, the primary objective of this study was to investigate differences in circulating IL-22 levels between patients with STEMI undergoing PPCI and healthy controls; the secondary objectives were to compare serum IL-22 levels considering the culprit coronary artery, door-to-balloon time (DBT), final angiographic result, CAD classification, and presence of diabetes mellitus (DM).

## 2. Materials and Methods

This cross-sectional, descriptive, and analytical study was conducted in a reference hospital for cardiology. A total of 210 patients with STEMI (symptoms plus ST elevation, new LBBB, or new RBBB) undergoing PPCI and 70 healthy controls (self-declared healthy volunteers, i.e., without disease to have normal circulating IL22) were recruited between January 2022 and August 2023.

The inclusion criteria were an age of ≥18 years and a clinical diagnosis of STEMI treated with PPCI. The exclusion criteria were previous or current oncologic disease, severe liver disease, blood dyscrasia, inability to respond to the questionnaire, or refusal to participate in the study. The controls were healthy volunteers who were ≥18 years old. 

**Figure 1:**
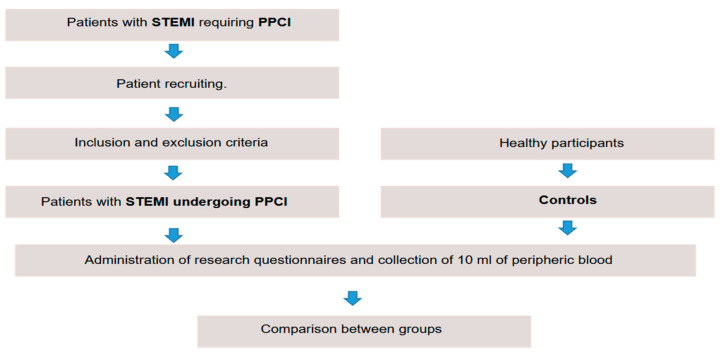
Flowchart of the study. Source: prepared by the author (2022).

Patients meeting these criteria were invited to participate in the study. Those who accepted signed an informed consent form (Figure 1).

After admission, the participants answered a clinical questionnaire. Clinical and socioeconomic parameters were self-declared and documented in a research-specific clinical record that was stored in a database.

The culprit vessel was defined as the coronary artery causing STEMI and undergoing PPCI. Multivessel CAD was defined as two or more obstructed vessels (≥70%), and single-vessel CAD was defined as only one vessel being affected. DBT was classified as >60 min or ≤60 min (according to the guidelines, up to 60 min is defined as the goal for ideal reperfusion). The final coronary flow was measured according to the criteria proposed by the Thrombolysis in Myocardial Infarction (TIMI) Study Group [14]. Angiographic success was determined by the TIMI 3 final coronary flow, a residual lesion of <10%, and the absence of thrombi and dissections.

### 2.1. Serum IL-22 Analysis

Commonly used materials, including a dry tube containing separating gel for obtaining and storing serum, one pair of procedure gloves, one 21 or 23 G scalp or a 30 × 06 or 30 × 07 needle, a 10 mL syringe, one tourniquet, two cotton balls, and 5 mL of 70% alcohol, were used to collect blood.

A large vein was identified for puncture and the subsequent collection of blood. The participant was instructed to lower their arm and open and close their hand. A gentle massage of the patient′s arm in the direction from the wrist to the elbow was performed, followed by palpation of the vein using the index finger (palpation with the thumb should be avoided). The vein was stabilized using the fingers in case of flaccidity. Touching the patient′s vein with the fingers for localization was contraindicated, as this could cause hemolysis and consequent changes.

The standard technique was used to collect 10 mL of peripheral blood for the measurement of IL-22 levels.

The blood was centrifuged, and the supernatant was collected and stored at −80 °C. A sandwiched enzyme-linked immunosorbent assay (ELISA) was used to quantify serum IL-22 levels using the Human IL-22 DuoSet^®^ kit (R&D Systems Inc., Minneapolis, MN, USA and CAN) according to the manufacturer′s recommendations. This kit had a minimum detection level of 50 pg/mL.

As all patients underwent PPCI, the timing of the blood sample collection was within 12 h after the index event and was performed electively in the control group.

### 2.2. Statistical Analysis

The data included a descriptive analysis of the clinical and angiographic characteristics of the patients and a comparative analysis of serum IL-22 levels between cases and controls. Numeric parameters were analyzed using the Shapiro–Wilk normality test. Normal parameters are presented as the mean and standard deviation, and non-normal parameters are presented as the median and 25th and 75th percentiles. Categorical parameters are presented as absolute values and percentages.

Student′s *t*-test, the Mann–Whitney test, the Kruskal–Wallis test, and analysis of variance (ANOVA) were used to analyze the numerical parameters depending on their normality. Categorical parameters were analyzed using the chi-square test or Fisher′s exact test. Statistical analyses were prepared by an independent statistician using the SPSS software version 23. *p*-values ≤ 0.05 were considered statistically significant.

All participants were comparatively analyzed. Subsequently, the cases were divided into five subgroups considering the culprit vessel, CAD classification, DBT, angiographic success of PPCI, and DM. These subgroups were compared.

### 2.3. Ethical Statement

This study was conducted according to ethical principles for clinical research and was approved by the ethical committee of the institution.

## 3. Results

This study included 280 participants: 210 patients (mean age: 63.5 ± 11.6 years old) with STEMI who underwent PPCI and 70 healthy controls (mean age: 54 ± 4.1 years old; men: 42 (60%); women: 28 (40%)). In the patient group, the descriptive analysis exhibited a higher prevalence of men (63.8% men, 36.2% women). The most prevalent risk factors were hypertension (76.2%), smoking (55.2%), and diabetes mellitus (38.1%) (Table 1).

In the subgroups, descriptive analysis showed that 83.3% of cases had a DBT of >60 min and that the anterior descending artery (LAD) was affected in 53.8% of cases. Multivessel CAD was present in 71.4% of cases, with an angiographic success rate of 84.8% (Table 2).

Serum IL-22 levels were lower in the cases 149.63 pg/mL (84.99–294.56) than in the controls 482 pg/mL (344.33–641.00), *p* < 0.001.

There were no clinical characteristic differences between patients when they were compared according to culprit artery, coronary artery disease extension, door-to-balloon time ≤60 vs. >60 min, angiographic success vs. unsuccess, and diabetics vs. non-diabetics.

Comparison of serum IL-22 levels according to subgroups: left anterior descending artery as culprit artery (*n* = 113 patients) 146 pg/mL (63.60–279.67) vs. left circumflex (*n* = 37 patients) 191.71 pg/mL (121.80–388.97) vs. right coronary artery (*n* = 80 patients) 144.57 pg/mL (70.84–242.43), *p* = 0.03; multiple vessels (*n* = 150 patients) 149.23 pg/mL (61.60–306.33) vs. single vessel (*n* = 60 patients) 153.79 pg/mL (101.00–271.33), *p* = 0.2; door-to-balloon time ≤60 min (*n* = 35 patients) 106 pg/mL (49.60–171.71) vs. >60 min (*n* = 175 patients) 153 pg/mL (88.86–313.60), *p* = 0.04; angiographic success (*n* = 178 patients) 149.67 pg/mL (84.99–281.33) vs. angiographic unsuccess (*n* = 32 patients) 137.36 pg/mL (84.13–346.33), *p* = 0.8; diabetics (*n* = 80 patients) 147.80 pg/mL (70.84–276.96) vs. non-diabetics (*n* = 130 patients) 151.33 pg/mL (85.90–306.33), *p* = 0.3.

## 4. Discussion

Herein, the results demonstrated lower circulating IL-22 in patients with STEMI undergoing PPCI than in healthy controls. In the subgroups, cases with a DBT of <60 min and with the RCA as the culprit artery had lower IL-22 levels. The CAD classification, angiography success, and DM showed no influence on IL-22 levels. 

Some studies indicated that high IL-22 levels have an atherogenic effect, especially in stable diseases. However, it is unclear if this is a specific cause or part of an immune–inflammatory response [15,16].

Conversely, animal studies had different results. IL-22 had a protective effect against myocardial remodeling and unfavorable cardiovascular outcomes after the induction of AMI in rats, which may have been linked to atheroma stabilization. Differently from most human studies, which evaluate CCS, that study analyzed an ACS model [17,18].

Yamamoto et al. [18] assessed the role of endogenous IL-22 in mice. In their study, they induced AMI by ligating the left coronary artery in wild-type and IL-22 knock-out mice, with a significantly lower survival rate in IL-22 knock-out animals due to a higher rate of cardiac rupture. Tang et al. [19] assessed the effect of exogenous IL-22 in rats. They induced AMI through ADA ligation, followed by the subcutaneous injection of 100 µg/kg/day of recombinant IL-22 (IL-22R) for seven days. Animals treated with IL-22R exhibited attenuated adverse ventricular remodeling and improved post-AMI cardiac function. It is noteworthy that the pharmacological inhibition of STAT3 also inhibited IL-22 [19].

Zhang et al. [17] evaluated 26 patients with non-ST-segment elevation myocardial infarction (NSTEMI), 16 patients with unstable angina, 16 patients with stable angina, and 16 healthy controls. They reported significantly higher plasma IL-22 levels in NSTEMI patients (33.09 ± 6.53 pg/mL) than in the patients with unstable angina (29.86 ± 3.49 pg/mL, *p* = 0.02), patients with stable angina (26.96 ± 3.09 pg/mL, *p* < 0.001), and healthy controls (24.16 ± 2.46 pg/mL, *p* < 0.001).

Unlike in the study by Zhang et al. [17], our study exhibited lower IL-22 levels in patients with STEMI who underwent PPCI. However, the clinical presentation and the treatment of our patients were different from those in their article (patients with STEMI who underwent PPCI vs. NSTEMI and CCS patients).

On the other hand, animal studies associated higher IL-22 levels with positive remodeling effects and increased survival, but the animals analyzed in those studies had no coronary reperfusion after AMI induction, which hindered comparisons with our model. The impact of coronary reperfusion on circulating IL-22 levels needs further clarification.

IL-22 is part of the IL-10 family and acts at a cellular level, mainly through the Jak/STAT pathway. It has a pro-atherogenic effect by inducing endothelial cells to produce ICAM-1 and VCAM-1 and stimulating monocyte recruitment [7,8]. STAT3 phosphorylation is induced after IL-22 binds with its receptors (IL-22R1 and IL-10R2) [8].

After activation, STAT3 translocates to the nucleus, modulating the expression of its target genes [20]. In humans, STAT3 pathway signaling directly relates to the expression of inflammatory mediators, which act in leukocyte activation and the progression of endothelial dysfunction [13]. In animals, blocking this pathway may be associated with improved clinical prognosis [13,19].

To summarize, IL-22 appeared to have an atherogenic effect in a stable coronary disease model in humans, and patients with NSTEMI had higher serum IL-22 levels than those of patients with CCS and healthy controls. In AMI-induced animal models, higher IL-22 levels were associated with better survival. This study confirmed that patients with STEMI undergoing PPCI had lower circulating IL-22 levels than those of healthy controls. 

In the case of STEMI and PPCI, acute arterial occlusion causes myocardial ischemia and necrosis, triggering an inflammatory cascade whose severity depends on the anatomical territories involved [6,15,21,22]. We found no studies in the literature on IL-22 levels in patients with STEMI undergoing PPCI.

DM is a well-known risk factor for CAD. It is associated with a pro-inflammatory status that accelerates the onset of atherosclerosis. It is also an independent predictor of worse prognosis in patients with ACS [23,24]. Despite this, IL-22 levels had no correlation with DM in this study.

The role of IL-22 in patients with DM and STEMI is not clear. However, we can infer a crosstalk between these mediators because IL-22 is part of the IL-10 family. IL-10 decreases inflammation, potentially improving the treatment of DM2 by inhibiting pro-inflammatory factors and increasing peripheral sensitivity to insulin [25]. Thus, new studies should investigate the synergism between IL-22 and IL-10 actions. 

Our study shows that CAD classification, angiographic success, and the presence of diabetes mellitus (DM) did not influence serum IL-22 levels. This is the first study to evaluate these issues in the described population. The absence of differences between these subgroups should be better understood, and additional studies are needed to clarify these results. A DBT of less than 60 min and RCA infarction are associated with a better prognosis. Our data revealed that the circulating IL 22 levels were lower in patients with a DBT of less than 60 min and with the RCA as the culprit artery, so it is possible that, in such patients, the lower levels of circulating IL 22 have a protective effect. However, we did not evaluate the prognosis, so this is only a suggested hypothesis.

Myocardial infarction related to RCA was considered for a long time to be less severe than left coronary artery infarction. Compared to left coronary artery, the RCA supplies less muscles.

We should understand that this information about RCA and IL22 is new, so it will be necessary to generate more data to define this issue. Additionally, this is a subgroup analysis, so the result is for hypothesis generation.

We know that sometimes we do not always thoroughly understand what happens, but knowledge should be built by adding new research on top of previous research. Our team is looking for new information, and we have a pipeline that will be developed. 

DBT less than 60 min is associated with less necrosis and may also be associated with a different pattern of inflammation.

Reperfusion may reduce the amount of ischemia and necrosis, so it is possible that the method of inflammation may be different. It is well defined that reperfusion changes the size of infarction. Additionally, there is no information about IL22 in patients with STEMI undergoing reperfusion through percutaneous coronary intervention. Another possibility is that the time of the blood sample was too early to identify the peak of IL22.

Considering the lack of data on IL-22 in some subgroups of STEMI patients undergoing PPCI, future studies are needed to confirm our finding that patients with a DBT of <60 min and with RCA as the culprit artery have lower IL-22 levels than those of the controls.

The main limitations of this study include its single-center design, the inclusion of patients treated for STEMI with medications that can affect IL-22 levels, the analysis of only a single IL-22 dosage, and the lack of evaluation of the association between IL-22 levels and prognosis due to the low rate of clinical events after PPCI. Additionally, there was no analysis between troponin and IL22.

Of note, medicines such as statins can change the circulating IL-22 levels, but these patients must be treated according to guidelines and recommendations. We should understand our findings according to this context, and it should be noted that the inflammation, immunological response, and molecules in this scenario are dynamic, and their effects depend on their interactions. Therefore, we aim to contribute to this field.

## 5. Conclusions

This study demonstrated that IL-22 levels were significantly lower in patients with STEMI than in healthy controls.

Within the subgroups, patients with a door-to-balloon time (DBT) of less than 60 min and those with the right coronary artery (RCA) as the culprit artery exhibited lower IL-22 levels. However, the CAD classification, angiography success, and presence of diabetes mellitus (DM) did not influence the serum IL-22 levels.

Additional research with follow-up will be useful in understanding the role of IL-22 in patients with acute myocardial infarction who underwent PPCI.

## Figures and Tables

**Table 1:** Clinical characteristics of the patients.

Variables	Patients = 210 (100.0)
Male, *n* (%)	134 (63.8)
Female, *n* (%)	76 (36.2)
Hypertension, *n* (%)	160 (76.2)
DM, *n* (%)	80 (38.1)
Smoking, *n* (%)	116 (55.2)
DLP, *n* (%)	54 (25.7)
Previous AMI, *n* (%)	37 (17.6)
Previous PCI, *n* (%)	17 (8.1)
Stroke, *n* (%)	8 (3.8)
CKD	7 (3.3)

DM: diabetes mellitus; DLP: dyslipidemia; AMI: acute myocardial infarction; PCI: percutaneous coronary intervention; CKD: chronic kidney disease.

**Table 2:** Door-to-balloon time, culprit artery, angiographic success, and coronary artery disease extension: descriptive analyses.

Patients	210 (100.0%)
DBT	
≤60 min, *n* (%)	35 (16.7)
60 min, *n* (%)	175 (83.3)
Culprit artery	
LCX, *n* (%)	37 (17.6)
LAD, *n* (%)	113 (53.8)
RCA, *n* (%)	60 (28.6)
Angiographic success	
No, *n* (%)	32 (15.2)
Yes, *n* (%)	178 (84.8)
CAD extension	
Multivessel, *n* (%)	150 (71.4)
Single-vessel, *n* (%)	60 (28.6)

DBT: door-to-balloon time, left circumflex artery (LCX), left anterior descending artery (LAD), right coronary artery (RCA), and coronary artery disease (CAD).

## Data Availability

The data generated and analyzed during this study are included in this published article and are available from the corresponding author.

## References

[1] Lloyd-Jones D., Adams R., Carnethon M., De Simone G., Ferguson T.B., Flegal K., Ford E., Furie K., Go A., Greenlund K. (2009). Heart disease and stroke statistics -2009 update: A report from the American Heart Association Statistics Committee and Stroke Statistics Subcommittee. Circulation.

[2] Libby P. (2013). Mechanisms of acute coronary syndromes and their implications for therapy. N. Engl. J. Med..

[3] Pober J.S., Sessa W.C. (2007). Evolving functions of endothelial cells in inflammation. Nat. Rev. Immunol..

[4] Vogel B., Claessen B.E., Arnold S.V., Chan D., Cohen D.J., Giannitsis E., Gibson C.M., Goto S., Katus H.A., Kerneis M. (2019). ST-segment elevation myocardial infarction. Nat. Rev. Dis. Primers.

[5] Bergmark B.A., Mathenge N., Merlini P.A., Lawrence-Wright M.B., Giugliano R.P. (2022). Acute coronary syndromes. Lancet.

[6] Loh J.P., Tan L.L., Zheng H., Lau Y.H., Chan S.P., Tan K.B., Chua T., Tan H.C., Foo D., Lee C.W. (2018). First Medical Contact-to-Device Time and Heart Failure Outcomes Among Patients Undergoing Primary Percutaneous Coronary Intervention. Circ. Cardiovasc. Qual. Outcomes.

[7] Luo J.W., Hu Y., Liu J., Yang H., Huang P. (2021). Interleukin-22: A potential therapeutic target in atherosclerosis. Mol. Med..

[8] Che Y., Su Z., Xia L. (2020). Effects of IL-22 on cardiovascular diseases. Int. Immunopharmacol..

[9] Rutz S., Eidenschenk C., Ouyang W. (2013). IL-22, not simply a Th17 cytokine. Immunol. Rev..

[10] Jones B.C., Logsdon N.J., Walter M.R. (2008). Structure of IL-22 bound to its high-affinity IL-22R1 chain. Structure.

[11] Mühl H., Bachmann M. (2019). IL-18/IL-18BP and IL-22/IL-22BP: Two interrelated couples with therapeutic potential. Cell. Signal..

[12] Camaré C., Pucelle M., Nègre-Salvayre A., Salvayre R. (2017). Angiogenesis in the atherosclerotic plaque. Redox Biol..

[13] Chen Q., Lv J., Yang W., Xu B., Wang Z., Yu Z., Wu J., Yang Y., Han Y. (2019). Targeted inhibition of STAT3 as a potential treatment strategy for atherosclerosis. Theranostics.

[14] Chesebro J.H., Knatterud G., Roberts R., Borer J., Cohen L.S., Dalen J., Dodge H.T., Francis C.K., Hillis D., Ludbrook P. (1987). Thrombolysis in Myocardial Infarction (TIMI) Trial, Phase I: A comparison between intravenous tissue plasminogen activator and intravenous streptokinase. Clinical findings through hospital discharge. Circulation.

[15] Torquati L., Coombes J.S., Murray L., Hasnain S.Z., Mallard A.R., McGuckin M.A., Fassett R.G., Croci I., Ramos J.S. (2019). Fibre Intake Is Independently Associated with Increased Circulating Interleukin-22 in Individuals with Metabolic Syndrome. Nutrients.

[16] Zhong Y., Tang R., Lu Y., Wang W., Xiao C., Meng T., Ao X., Li X., Peng L., Kwadwo Nuro-Gyina P. (2020). Irbesartan may relieve renal injury by suppressing Th22 cells chemotaxis and infiltration in Ang II-induced hypertension. Int. Immunopharmacol..

[17] Zhang L., Wang T., Wang X.Q., Du R.Z., Zhang K.N., Liu X.G., Ma D.X., Yu S., Su G.H., Li Z.H. (2013). Elevated frequencies of circulating Th22 cell in addition to Th17 cell and Th17/Th1 cell in patients with acute coronary syndrome. PLoS ONE.

[18] Yamamoto M., Yasukawa H., Takahashi J., Nohara S., Sasaki T., Shibao K., Akagaki D., Okabe K., Yanai T., Shibata T. (2023). Endogenous interleukin-22 prevents cardiac rupture after myocardial infarction in mice. PLoS ONE.

[19] Tang T.T., Li Y.Y., Li J.J., Wang K., Han Y., Dong W.Y., Zhu Z.F., Xia N., Nie S.F., Zhang M. (2018). Liver-heart crosstalk controls IL-22 activity in cardiac protection after myocardial infarction. Theranostics.

[20] Linton M.F., Moslehi J.J., Babaev V.R. (2019). Akt Signaling in Macrophage Polarization, Survival, and Atherosclerosis. Int. J. Mol. Sci..

[21] Fazel R., Joseph T.I., Sankardas M.A., Pinto D.S., Yeh R.W., Kumbhani D.J., Nallamothu B.K. (2020). Comparison of Reperfusion Strategies for ST-Segment-Elevation Myocardial Infarction: A Multivariate Network Meta-analysis. J. Am. Heart Assoc..

[22] Park D.W., Clare R.M., Schulte P.J., Pieper K.S., Shaw L.K., Califf R.M., Ohman E.M., Van de Werf F., Hirji S., Harrington R.A. (2014). Extent, location, and clinical significance of non-infarct-related coronary artery disease among patients with ST-elevation myocardial infarction. JAMA.

[23] Faludi A.A., Izar M.C.O., Saraiva J.F.K., Chacra A.P.M., Bianco H.T., Afiune A., Neto A.A., Bertolami A., Pereira A.C., Lottenberg A.M. (2017). Atualização da Diretriz Brasileira de Dislipidemias e Prevenção da Aterosclerose—2017. Arq. Bras. Cardiol..

[24] Jin Z., Zhang Q., Liu K., Wang S., Yan Y., Zhang B., Zhao L. (2024). The association between interleukin family and diabetes mellitus and its complications: An overview of systematic reviews and meta-analyses. Diabetes Res. Clin. Pract..

[25] Hong E.G., Ko H.J., Cho Y.R., Kim H.J., Ma Z., Yu T.Y., Friedline R.H., Kurt-Jones E., Finberg R., Fischer M.A. (2009). Interleukin-10 prevents diet-induced insulin resistance by attenuating macrophage and cytokine response in skeletal muscle. Diabetes.

